# Implementation of Artificial Synapse Using IGZO-Based Resistive Switching Device

**DOI:** 10.3390/ma17020481

**Published:** 2024-01-19

**Authors:** Seongmin Kim, Dongyeol Ju, Sungjun Kim

**Affiliations:** Division of Electronics and Electrical Engineering, Dongguk University, Seoul 04620, Republic of Koreajudongyeol0117@gmail.com (D.J.)

**Keywords:** RRAM, IGZO, synapse, resistive switching, SRDP, STDP

## Abstract

In this study, we present the resistive switching characteristics and the emulation of a biological synapse using the ITO/IGZO/TaN device. The device demonstrates efficient energy consumption, featuring low current resistive switching with minimal set and reset voltages. Furthermore, we establish that the device exhibits typical bipolar resistive switching with the coexistence of non-volatile and volatile memory properties by controlling the compliance during resistive switching phenomena. Utilizing the IGZO-based RRAM device with an appropriate pulse scheme, we emulate a biological synapse based on its electrical properties. Our assessments include potentiation and depression, a pattern recognition system based on neural networks, paired-pulse facilitation, excitatory post-synaptic current, and spike-amplitude dependent plasticity. These assessments confirm the device’s effective emulation of a biological synapse, incorporating both volatile and non-volatile functions. Furthermore, through spike-rate dependent plasticity and spike-timing dependent plasticity of the Hebbian learning rules, high-order synapse imitation was done.

## 1. Introduction

Currently, due to limitations in speed and efficiency, traditional Von-Neumann computing architectures are facing challenges. The computing architecture of Von Neumann, which is based on the physical separation of the computer processing unit and memory unit, encounters a bottleneck due to the differing processing speeds between the two units [1,2]. To overcome these limitations, there is a rapidly evolving paradigm known as neuromorphic computing, inspired by the structure and functions of our biological brain [3]. The neural information processing of our brain occurs based on the firing of spikes from neurons to neurons, and synapses to synapses, making the process fast and energy efficient [4]. Thus, the adapting of neuromorphic computing aims to meet the demands of parallel signal processing, high power efficiency, and rapid complex task computation [5,6,7,8]. Within the realm of neuromorphic computing, electronic synapse devices play a pivotal role as efficient hardware components.

In the context of neuromorphic computing, where a high synaptic density is essential, emulating a single synapse using multiple transistors and capacitors is not an efficient approach [9]. Instead, resistive random-access memory (ReRAM) or memristors have emerged as promising candidates for artificial synapses. They exhibit a simple two-terminal structure, low-power consumption, and exceptional durability in switching, making them highly suitable for this purpose [10,11,12,13,14]. The act of data storing in RRAM occurs based on an event called the resistive switching phenomena, where under an applied bias, the internal resistance state of RRAM device switches between ‘On’ and ‘Off’ [15]. This phenomenon of switching takes place within the insulating layer of RRAM, positioned between two electrodes. Recent studies have documented the utilization of diverse materials like polymers, 2D materials, such as MoS2 and H-BN, as well as perovskites for the insulating film in RRAM devices [3,16,17,18,19]. However, RRAMs employing transition metal oxides (TMO) as the insulating material have been the focus of widespread and predominant research due to the simplicity of the material and good compatibility with the silicon CMOS fabrication process [20,21,22,23,24,25]. As one of the TMOs, Indium gallium zinc oxide (IGZO) holds particular significance in this field. IGZO is utilized as the channel material in thin-film transistors (TFTs), playing a crucial role in regulating electrical signals and controlling transistor operations [26,27,28]. Its unique properties enable IGZO to achieve multistage intermediate resistance states, mimicking various switching states through the manipulation of oxygen vacancies within its spatial structure [29]. Furthermore, the fine-tuning of these intermediate states allows IGZO-based memristors to demonstrate outstanding durability and consistent switching voltages, facilitating the simulation of critical synaptic functions, including learning experiences [30,31,32,33].

In this study, a device with an indium tin oxide (ITO)/IGZO/TaN structure was fabricated, and various synaptic functions, including potentiation and depression, paired-pulse facilitation (PPF), excitatory postsynaptic currents (EPSC), spike-rate dependent plasticity (SRDP), and spike-timing-dependent plasticity (STDP), were evaluated and analyzed. Additionally, during the resistive switching phenomenon, by simply controlling the compliance current (CC), the coexistence of non-volatile and volatile memory characteristics of the memristor device is depicted. The material characteristics of the device were further investigated through X-ray photoelectron spectroscopy (XPS) and transmission electron microscopy (TEM). Additionally, the study incorporated the MNIST pattern recognition system validation to assess the device’s performance. The IGZO-based memristor we created demonstrated various synaptic functions, surpassing those outlined in previous reports, as detailed in Table 1.

## 2. Materials and Methods

The ITO/IGZO/TaN device was fabricated through the following process. First, a 100-nm-thick TaN as the bottom electrode was deposited on a commercially available SiO_2_/Si using an RF reactive sputtering. The pressure in the main chamber was maintained at 3 mTorr with a gas mixture of Ar (19 sccm) and N_2_ (1 sccm) used under 150 W RF power. Then, the insulating 30-nm-thick IGZO film was deposited on the TaN bottom electrode using RF reactive sputtering, using a gas mixture of Ar (10 sccm) and O_2_ (2 sccm). A target of IGZO with an elemental composition of In, Ga, and Zn in a ratio of 1:1:1 was employed, and it had a purity level of 99.99%. The power and the pressure of the main chamber were 100 W and 3 mTorr. Then, using photolithography, a square pattern with a pattern size of 100 µm × 100 µm was obtained. Finally, by RF reactive sputtering, a 100-nm-thick ITO top electrode was acquired. The Ar gas of 8 sccm with power and pressure of 60 W, 3 mTorr were needed. The electrical characteristics of fabricated ITO/IGZO/TaN was determined through Keithley 4200-SCS semiconductor parameter analyzer (Keithley 4200-SCS and PMU ultrafast mode, Tektronix Inc., Beaverton, OR, USA) and a 4225-PMU pulse measuring unit (Keithley 4200-SCS and PMU ultrafast mode, Tektronix Inc., Beaverton, OR, USA). The bias was applied to the top electrode ITO, with a grounded TaN bottom electrode. Additionally, the structural and chemical properties of the ITO/IGZO/TaN device were proved through the cross-sectional image of transmission electron microscope (TEM, KANC, Suwon 16229, Republic of Korea) and X-ray photoelectron (XPS) analysis.

## 3. Results and Discussion

### 3.1. Structural Element Analysis of IGZO-Based Memristor

**Figure 1:**
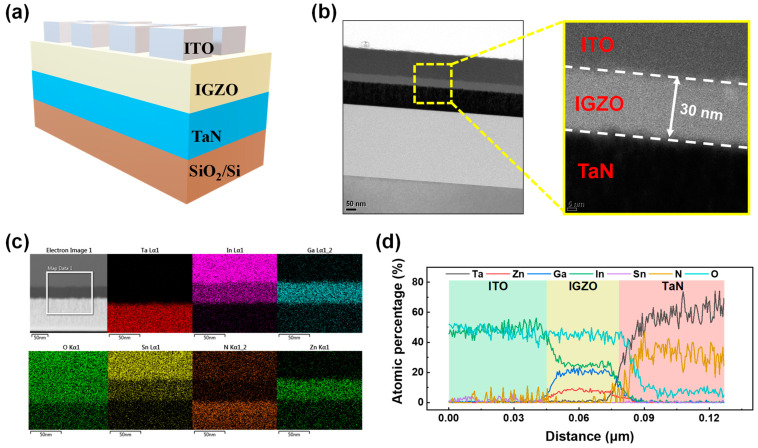
(**a**) Schematic illustration of ITO/IGZO/TaN device. (**b**) Cross-sectional TEM image of ITO/IGZO/TaN device. (**c**) EDS color mapping of elements In, Sn, Ta, Ga, O, N, and Zn. (**d**) Atomic percentage spectra of ITO/IGZO/TaN device.

Figure 1a depicts a schematic of the ITO/IGZO/TaN device, while Figure 1b shows a cross-sectional TEM image. The high-resolution TEM image reveals 100 nm thick ITO and TaN electrodes, separated by a 30 nm IGZO insulating layer. Figure 1c presents an EDS color mapping of ITO/IGZO/TaN, displaying the elemental distribution of In, Sn, Ta, O, N, Zn, and Ga, confirming the chemical composition of each layer. Additionally, the atomic percentage ratio of these elements, measured at various distances from the top electrode, showcases the composition of the ITO, IGZO, and TaN layers.

**Figure 2:**
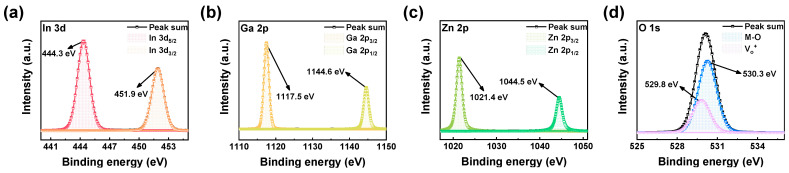
XPS spectra of (**a**) In 3d, (**b**) Ga 2p, (**c**) Zn 2p, and (**d**) O 1s.

In Figure 2, the chemical composition of the insulating IGZO layer was investigated using XPS analysis in depth mode, gradually etching layer by layer. The spectrum exhibits peaks attributed to elements In, Ga, Zn, and O. In Figure 2a, the In 3d spectrum of the IGZO film reveals two peaks, In 3d_5/2_ and In 3d_3/2_, at approximately 444.3 eV and 451.9 eV binding energies, respectively. Figure 2b illustrates the Ga 2p spectrum, displaying double peaks, Ga 2p_3/2_ and Ga 2p_1/2_, situated at binding energies of 1117.5 eV and 1144.6 eV. Furthermore, Figure 2c presents the Zn 2p spectrum, showcasing two peaks, Zn 3p_3/2_ and Zn 3p_1/2_, located at binding energies of 1021.4 eV and 1044.5 eV [40]. Moving on to Figure 2d, the O 1s spectrum reveals a peak at 530.3 eV, indicating metal–oxygen bonding and confirming the presence of an insulating oxygen-rich IGZO film. Additionally, a second peak at 529.8 eV signifies the existence of oxygen vacancies (defects) within the IGZO film [25].

### 3.2. Electrical Characteristics and Conduction Mechanism of IGZO-Based Memristor

**Figure 3:**
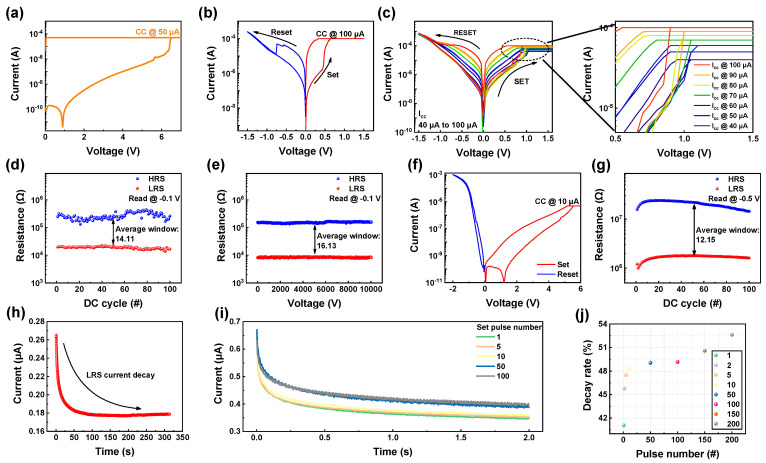
(**a**) Forming process of ITO/IGZO/TaN device. (**b**) Typical I-V curve of non-volatile switching ITO/IGZO/TaN device. (**c**) Multi-level characteristics of ITO/IGZO/TaN device gained by differing CC. (**d**) DC endurance uniformity during 10^2^ cycles of non-volatile switching ITO/IGZO/TaN device. (**e**) Retention during 10^4^ s of non-volatile switching ITO/IGZO/TaN device. (**f**) Typical I-V curve of volatile switching ITO/IGZO/TaN device. (**g**) DC endurance uniformity during 10^2^ cycles of volatile switching ITO/IGZO/TaN device. (**h**) LRS current decay observed from volatile switching ITO/IGZO/TaN device for 320 s. (**i**) Current decay controlled by varying set pulse numbers. (**j**) Decay rate illustrated as a function of pulse number.

In Figure 3, the electrical characteristics of the ITO/IGZO/TaN device are explored. Prior to the resistive switching process, an initial breakdown or forming process is carried out to establish the conductive pathway necessary for non-volatile resistive switching [41]. Figure 3a demonstrates the completion of this forming process by applying a forming voltage of 6.5 V and a compliance current (CC) of 50 µA. The CC is employed during bias application to prevent hard breakdown, which could otherwise damage the device. This controlled application of CC prevents a sudden increase in current, thereby restricting the width of the conductive filament and aiding the resistive switching properties of the device. Figure 3b presents the typical I–V curve illustrating the bi-polar resistive switching of the ITO/IGZO/TaN device, showcasing set, and reset processes occurring at opposite biases. During the set process, a bias of 1.5 V and a CC of 100 µA are applied, leading to an increase in current and a transition from a high resistance state (HRS) to a low resistance state (LRS). Conversely, applying an opposite bias of −1.5 V induces a decrease in current, switching the device from HRS to LRS. In a RRAM device, a multi-level characteristic is important, capable of enhancing storage density which is related to the cost effectiveness [42,43,44,45]. Storing data solely in HRS and LRS in RRAM can be expanded by incorporating multi-level characteristics, enabling the device to store data across a range between LRS and HRS. One of the ways to obtain multi-level characteristic is by controlling the CC during set process. As shown in Figure 3c, by varying the CC from 40 µA to 100 µA, seven different LRS are gained, while maintaining the same amount of HRS. This could stem from the conductive filament’s attributes. As stated, the CC governs the breadth of the conductive filament when a bias is applied. Thus, by regulating the path width linking the top and bottom electrodes, the volume of current passing through fluctuates accordingly. Hence, applying a substantial CC lead to the formation of a broad conductive filament, facilitating a substantial current flow. Conversely, applying a smaller CC results in the formation of a narrower conductive path, restricting the current flow and reducing the LRS current. The uniformity of HRS and LRS during continuous resistive switching is evaluated through DC endurance and retention tests. Figure 3d illustrates the stability of LRS and HRS after 10^2^ repeated set and reset cycles, showing an average window (HRS/LRS) of 14.11 without resistance state breakdown. Additionally, retention over 10^4^ s (Figure 3e) indicates no degradation in resistance states, maintaining an average window of 16.13, showcasing favorable non-volatile memory characteristic. Different from the non-volatile resistive switching, the volatile switching of the ITO/IGZO/TaN device can be observed by applying low CC during the forming process. As depicted in Figure 3f, when applied with a CC of 10 µA, volatile resistive switching is observed. Its repeatability and uniformity were tested by repeating the set and reset process for 10^2^ cycles, as depicted in Figure 3g. The average window of 12.15 was gained when read from the read voltage of −0.5 V. The volatile property of ITO/IGZO/TAN is illustrated in Figure 3h, where during retention for 320 s after the set process, the current decay of LRS was found. This may be due to the low CC generating narrow conductive filament, making it rupture under removed applied bias. By applying electrical pulse stimuli, this current decay property of ITO/IGZO/TaN device can be controlled, as illustrated in Figure 3i. To switch the resistance state of ITO/IGZO/TaN device from HRS to LRS, set pulse of 4 V, 10 ms was applied. When a single set pulse is applied, decay of current, returning to HRS is observed. However, by increasing the number of set pulses, the current decay decreases, gaining a higher current level. The rate of decay was then figured in Figure 3j, where the term decay rate was gained through the following formula shown in Equation (1):

(1) $$Decay\;rate\;\left(\%\right)=\frac{I_{d}}{I_{max}}\times 100$$

The terms I_d_, I_max_ refer to the current right after set pulse application and decay. As the set pulse number increases, the decay rate increases, implying that the volatile property of the ITO/IGZO/TaN device can be controlled by varying electrical stimuli.

**Figure 4:**
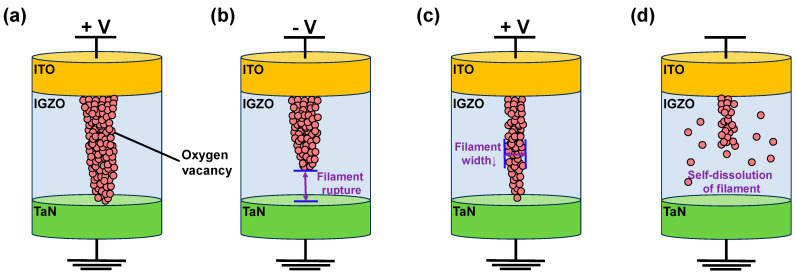
Conduction mechanism of non-volatile switching ITO/IGZO/TaN (**a**) set, and (**b**) reset. Conduction mechanism of volatile switching ITO/IGZO/TaN, (**c**) set, and (**d**) reset.

Conduction mechanisms of non-volatile and volatile switching characteristics of ITO/IGZO/TaN device are depicted in Figure 4. Previous studies reported that the RRAM device based on transition metal oxides switches due to the formation and rupture of conductive filament, where a set voltage generated a conductive pathway connecting the top and bottom electrode, allowing large current flow. On the other hand, by applying reverse bias, the conductive pathway constructed of defects ruptures, limiting the current flow [20,46,47,48]. Furthermore, previous studies of IGZO-based memristor reported the formation and rupture of conductive filament becoming the main cause of resistive switching [49,50,51]. Similarly, ITO/IGZO/TaN device switches based on the formation and rupture of conductive filament. Under positive bias application toward the top electrode, the oxygen ions migrate towards the top electrode under the formed electric field, leaving oxygen vacancies. The left oxygen vacancies accumulate and form a conductive filament, connecting the ITO and TaN electrode, switching the device from HRS to LRS (Figure 4a). Conversely, when negative bias is applied to the ITO electrode, the migrated oxygen ions are repelled back towards its original region, recombining with oxygen vacancies. Thus, the conductive filament ruptures, switching the device from LRS to HRS (Figure 4b). In the case of volatile switching in the ITO/IGZO/TaN device, the low CC applied during the resistive switching restricts the widening of conductive filament, occurring temporal resistance state change from HRS to LRS (Figure 4c). However, the narrow filament ruptures due to self-dissolution of oxygen vacancy when the applied bias is removed, changing the resistance state back to HRS (Figure 4d).

### 3.3. Synaptic Functions of IGZO-Based Memristor

**Figure 5:**
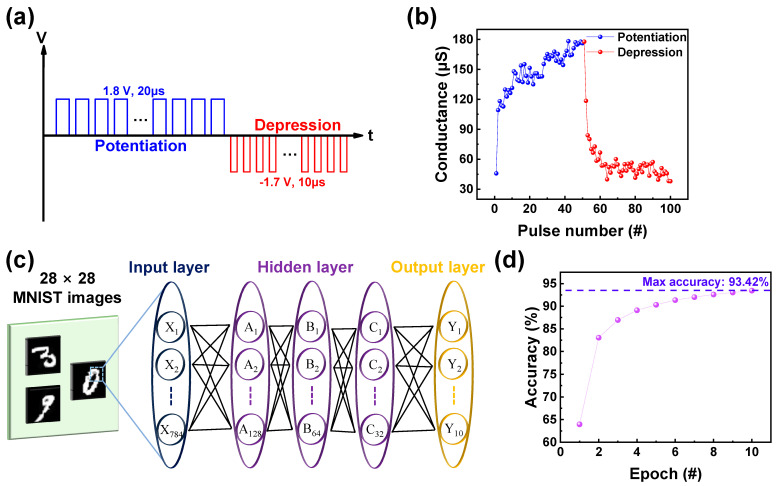
(**a**) Schematic illustration of pulse scheme used to acquire potentiation and depression. (**b**) Potentiation and depression curve of ITO/IGZO/TaN. (**c**) The framework of neural network-based PRS. (**d**) The pattern recognition accuracy of ITO/IGZO/TaN device gained through 10 consecutive epochs.

The synaptic properties of the ITO/IGZO/TaN device were investigated using identical pulse applications. The function of potentiation and depression refers to the strengthening and weakening of synaptic connections, enhancing the communication between neurons. The schematic illustration of the pulse scheme used to acquire potentiation and depression is illustrated in Figure 5a. To acquire a gradual increase and decrease in conductance, 50 consecutive set pulses, followed by 50 consecutive reset pulses were applied to the ITO/IGZO/TaN device [21]. The amplitude and width of the set and reset pulses are 1.8 V, 20 µs, and −1.7 V, 10 µs, respectively. Additionally, each pulse was followed by a read pulse of −0.1 V, to observe the change of conductance. The result of the applied pulse scheme is shown in Figure 5b, where potentiation and depression behavior, the increase and decrease in conductance value from 40 µs to 180 µs can be observed. For applications of replicating neural network models and parallel processing ability of capability of handling complex tasks, neural network based Modified National Institute of Standards and Technology (MNIST) pattern recognition system (PRS) was tested using Python in Google Colab. As shown in Figure 5c, the neural network consists of the input, hidden, and output layers, where the hidden layer specifically has three layers consequently, with each layer having 128, 64, and 32 nodes. For the training, 28 × 28-pixel handwritten number images converted from the potentiation and depression graph of Figure 4b were used. Prior to sequential training the linearity of the potentiation and depression curve was calculated using the following Equation (2) [52]:

(2) $$G={\left\{\left(G_{LRS}^{\alpha }-G_{HRS}^{\alpha }\right)\times \omega +G_{HRS}^{\alpha }\right\}}^{\frac{1}{\alpha }}\;if\;\alpha \;\neq \;0$$

where *ω* is a value between 0 and 1, and *α* is a value of linearity at that point. The equation results in different responses: for α > 1, it shows a concave-down response; for α < 1, it demonstrates a concave-up response, and for α = 1, it yields a perfectly linear response [53]. The conversion of data to handwritten numbers occurred through the increase and decrease in pixel values compared to the preceding image, following the pattern of potentiation or depression, each conductance state being utilized as synaptic weight. For the training and testing process, 50,000 and 10,000 random images were utilized, with the normalization process of each conductance state being done through the following Equation (3) [54]:

(3) $$G=\frac{G-G_{min}}{G_{max}-G_{min}}$$

The result of accuracy gained through 10 consecutive epochs is illustrated in Figure 5d, with the highest accuracy of 93.42% represented.

**Figure 6:**
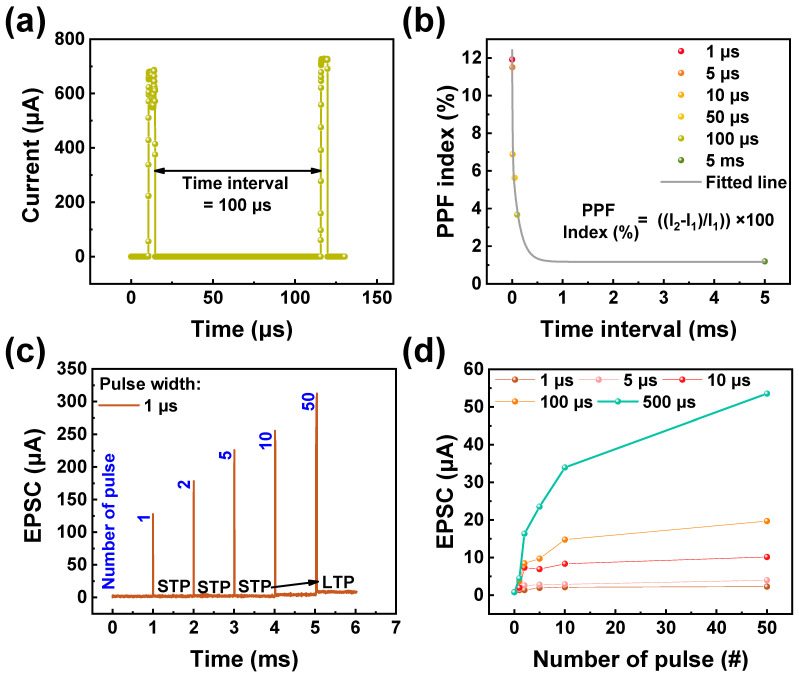
(**a**) PPF characteristic of ITO/IGZO/TaN device with a time interval of 100 µs. (**b**) Distribution of PPF as a function of the time interval. (**c**) The transition of STM to LTM gained through consecutive pulse applications. (**d**) EPSC variance acquired under varied pulse width conditions.

Next, the short-term synapse characteristic of ITO/IGZO/TaN was tested. Short-term memory (STM) is a part of the biological brain’s memory, which performs critical functions such as fast response and information filtering [4]. Under minor stimuli, a previous study reported the existence of STM, which may be due to the partial formation of conductive filament [55]. Similarly, the STM of ITO/IGZO/TaN was investigated with PPF as shown in Figure 6a,b. PPF is a function acquired by applying twin pulses to the device under different conditions. Due to the volatile behavior of the device, as the time interval increases, the current response of the second pulse will decrease, which can be interpreted as the forgetting process of the biological brain [56,57]. To acquire PPF behavior in the ITO/IGZO/TaN device, a twin pulse of 1.5 V, 5 µs was applied with intervals varying from 1 µs to 5 ms. The result of the applied twin pulse is depicted in Figure 6b, where it is observed that after a sufficient time interval, the PPF index of ITO/IGZO/TaN device decreases, favorably emulating STM. The PPF index is defined as Equation (4), where the terms I_2_ and I_1_ refer to the current response after the second and first pulse.

(4) $$\mathrm{PPF}\;\mathrm{index}\;\left(\%\right)=\frac{\left(I_{2}-I_{1}\right)}{I_{1}}\times 100$$

Furthermore, in the biological brain, the conversion from STM to long-term memory (LTM), may occur under the rehearsal of certain processes [58]. To obtain this conversion, excitatory postsynaptic current (EPSC) was tested with varied set pulse numbers of 1, 2, 5, 10, and 50 [59]. EPSC represents the flow of ions through the postsynaptic membrane in response to the neurotransmitter release, contributing to the excitatory signal transmission between neurons. For EPSC, a set pulse of 1.8 V, 1 µs was used with the read pulse of −0.1 V, 1 ms following to seek current change. The result is shown in Figure 6c, where for a small number of set pulses applied to the device (1, 2, 5, and 10), no significant change of current is observed if the change rate is low, resulting in a decay of the current, showing short-term potentiation (STP). On the other hand, after 50 consecutive set pulse applications, the read current of the ITO/IGZO/TaN device increases significantly, showing long-term potentiation (LTP). Furthermore, to observe the width-dependent EPSC response, the same test done under varied pulse width is illustrated in Figure 6d. The pulse width varied from 1 µs to 500 µs, with a fixed set pulse number. As a result, the device showed good output current–input pulse relationship, with an increasing EPSC response under bigger pulse width, accurately mimicking biological brain where bigger stimuli result in a bigger synaptic weight output [60].

**Figure 7:**
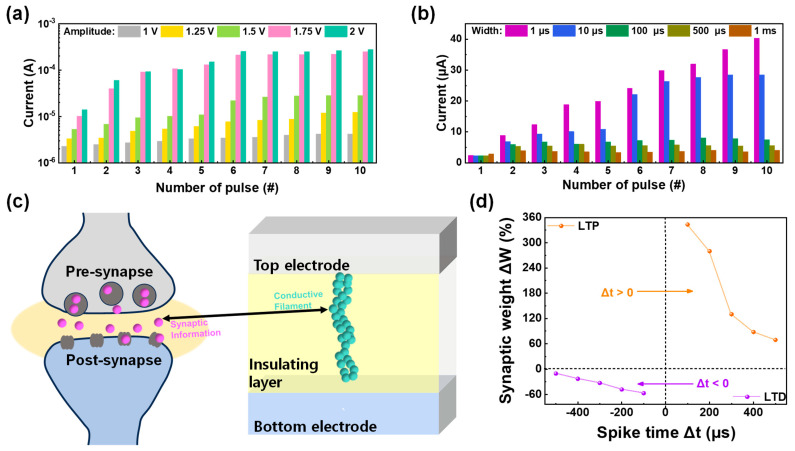
(**a**) The current response of SRDP as a function of pulse number. (**b**) The current response of SADP as a function of pulse number. (**c**) Schematic illustration of the biological brain’s pre- and post-synapse emulated in RRAM device. (**d**) The result of STDP as a function of spike time.

Next, some high-order synaptic plasticity was figured through Hebbian learning rules [61,62,63]. The Hebbian learning rule is a principle employed in synaptic devices and neural network models to elucidate how synapses connecting neurons can be modified in strength, either reinforced or weakened, depending on the simultaneous activation of these neurons. One of these learning rules is spike-rate dependent plasticity (SRDP), where the relationship between the output current and time interval between applied pulses is figured [64]. SRDP refers to changes in synaptic strength based on the firing rates of neurons. It plays a role in regulating the overall stability and adaptability of neural networks. A sequence of 10 successive set pulses was administered to the ITO/IGZO/TaN device, each pulse maintaining a consistent amplitude and duration of 1.5 V and 10 µs, respectively. However, the pulse interval varied between 1 µs and 1 ms to mimic the different spike firing rates of the synapse. The result of this pulse application is depicted in Figure 7a, and the interval-dependent current response shows that in a short time interval, the output rapidly increases. Additionally, to figure the spike strength-dependent plasticity of the ITO/IGZO/TaN device, spike-amplitude-dependent plasticity (SADP) was tested by applying ten sequential set pulse with varied pulse amplitude. In this case, the pulse interval and width are fixed at 10 µs, with set pulse amplitude varying from 1 to 2 V. The result of amplitude varied pulse application is illustrated in Figure 7b, with stronger stimuli resulting larger current is observed. Finally, the spike-timing dependent plasticity (STDP) was investigated to fully emulate synapse functions in the biological brain. In the biological brain, synaptic information moves through certain routes, which is called a synapse. Under certain synaptic weights, the activities of synapses are determined, resulting in a migration of information from pre- and post-synapse [46]. STDP involves modifications in synaptic strength based on the relative timing of spikes in the pre- and post-synaptic neurons. This mechanism is critical for temporal aspects of learning and memory. The RRAM device, which has two terminals, can efficiently emulate the biological synapse with each top and bottom electrode representing the pre- and post-synapse, as illustrated in Figure 7c. Additionally, the migration of synaptic information can be interpreted as the flow of current through the conductive filament formed in the insulating layer. The STDP behavior can be divided into two parts: long-term potentiation (LTP) and long-term depression (LTD) depending on the spike firing time (Δt = t_pre_ − t_post_). When the pre-synapse exceeds the post-synapse (Δt > 0), a positive set of pulses is applied to the device, resulting in increased synaptic weight (ΔW) and LTP. On the other hand, when post-synapse exceeds the pre-synapse (Δt < 0), a negative set of pulses is applied to the device, resulting in a decreased synaptic weight and LTD. The STDP of ITO/IGZO/TaN is shown in Figure 7d, where the term synaptic weight is defined as Equation (5):

(5) $$\Delta W\;\left(\%\right)=\frac{G_{f}-G_{i}}{G_{i}}\times 100$$

where G_f_ and G_i_ refer to the conductance values after and before pulse application, respectively. The LTP and LTD behavior is shown with synaptic weight varying under spike time and the synapse emulation with various functions are shown.

## 4. Conclusions

The resistive switching characteristics with various synaptic functions of IGZO-based RRAM device was investigated in detail. The resistive switching of the ITO/IGZO/TaN device occurred based on the formation and rupture of oxygen vacancy accumulated conductive filament in the insulating IGZO layer under migration of oxygen ions. Due to its low operating current and voltage, the device has favorable energy efficient applications. Furthermore, through various pulse applications, long-term and short-term synaptic functions, potentiation and depression, PPF, EPSC, SRDP, SADP and STDP with various pulse conditions were tested to effectively implement the artificial synapse.

## Figures and Tables

**Table 1:** Differences in characteristics between this study and previously reported IGZO-based synaptic devices.

No	Stack	Operating Voltage	Synaptic Functions	Ref.
1	Mo/a-IGZO/Ti/MO	−2 V~2 V	Potentiation, depression	[34]
2	Mo/IGZO/Mo	−2.5 V~2.5 V	Potentiation, depression	[35]
3	Ag/IGZO/TiN	−2 V~2.5 V	Potentiation, depression, STDP	[36]
4	Au/IGZO/Pt	−1 V~0.8 V	Potentiation, depression	[37]
5	Ti/TaO_x_/IGZO/Pt	−1.5 V~1.5 V	Potentiation, depression, PPF, SRDP	[38]
6	Ti/IGZO/Ti	−3 V~3 V	Potentiation, depression	[39]
7	ITO/IGZO/TaN	−1.5 V~1.5 V	Potentiation, depression, PPF, EPSC, SRDP, STDP	This work

## Data Availability

Data are contained within the article.
